# Supplementary material for: Modeling glioblastoma heterogeneity as a dynamic network of cell states
Source: Mol Syst Biol. 2021 Sep 16;17(9):e10105. doi: 10.15252/msb.202010105 (PMC8444284; doi:10.15252/msb.202010105)
Supplement: Supplementary file 5 — Source Data for Figure 3 [file MSB-17-e10105-s001.zip › Figure3A_sourcedata/GSEA_3065/nosigdb_state6.GseaPreranked.1622718443993/RICHARDS_ZHONG_NATURE_2018_NPCS_UPREG.html]

Details for gene set RICHARDS\_ZHONG\_NATURE\_2018\_NPCS\_UPREG[GSEA]

|  || Dataset | state6 |
| Phenotype | NoPhenotypeAvailable |
| Upregulated in class | na\_neg |
| GeneSet | RICHARDS\_ZHONG\_NATURE\_2018\_NPCS\_UPREG |
| Enrichment Score (ES) | -0.59923756 |
| Normalized Enrichment Score (NES) | -2.4638295 |
| Nominal p-value | 0.0 |
| FDR q-value | 0.0 |
| FWER p-Value | 0.0 |
Table: GSEA Results Summary

  

Fig 1: Enrichment plot: RICHARDS\_ZHONG\_NATURE\_2018\_NPCS\_UPREG      
 Profile of the Running ES Score & Positions of GeneSet Members on the Rank Ordered List

  

| PROBE | GENE SYMBOL | GENE\_TITLE | RANK IN GENE LIST | RANK METRIC SCORE | RUNNING ES | CORE ENRICHMENT || 1 | RPS27L |  |  | 91 | 0.218 | 0.0095 | No |
| 2 | SOX9 |  |  | 148 | 0.193 | 0.0208 | No |
| 3 | GNG5 |  |  | 326 | 0.147 | 0.0139 | No |
| 4 | HMGN2 |  |  | 679 | 0.108 | -0.0168 | No |
| 5 | H2AFV |  |  | 782 | 0.100 | -0.0194 | No |
| 6 | CREB5 |  |  | 1231 | 0.074 | -0.0642 | No |
| 7 | BTG3 |  |  | 2012 | 0.047 | -0.1498 | No |
| 8 | UCP2 |  |  | 2041 | 0.047 | -0.1488 | No |
| 9 | TUBA1B |  |  | 2087 | 0.045 | -0.1498 | No |
| 10 | HJURP |  |  | 2286 | 0.039 | -0.1691 | No |
| 11 | PTTG1 |  |  | 2304 | 0.038 | -0.1675 | No |
| 12 | MCM6 |  |  | 2484 | 0.034 | -0.1850 | No |
| 13 | RPA3 |  |  | 2563 | 0.032 | -0.1911 | No |
| 14 | ANP32E |  |  | 2570 | 0.032 | -0.1889 | No |
| 15 | SFRP1 |  |  | 2812 | 0.026 | -0.2143 | No |
| 16 | VIM |  |  | 2831 | 0.026 | -0.2140 | No |
| 17 | TMPO |  |  | 2978 | 0.022 | -0.2288 | No |
| 18 | CKS1B |  |  | 2985 | 0.022 | -0.2274 | No |
| 19 | WDR34 |  |  | 3222 | 0.017 | -0.2530 | No |
| 20 | DTYMK |  |  | 3271 | 0.016 | -0.2571 | No |
| 21 | HMGB3 |  |  | 3377 | 0.014 | -0.2679 | No |
| 22 | NOTCH1 |  |  | 3459 | 0.012 | -0.2762 | No |
| 23 | HSPB1 |  |  | 3587 | 0.010 | -0.2900 | No |
| 24 | DUT |  |  | 3596 | 0.009 | -0.2900 | No |
| 25 | TGIF1 |  |  | 3672 | 0.007 | -0.2980 | No |
| 26 | CENPW |  |  | 3936 | 0.002 | -0.3281 | No |
| 27 | CDC20 |  |  | 3996 | 0.001 | -0.3349 | No |
| 28 | ID4 |  |  | 4102 | -0.001 | -0.3469 | No |
| 29 | MDK |  |  | 4311 | -0.005 | -0.3704 | No |
| 30 | SNRPB |  |  | 4387 | -0.007 | -0.3784 | No |
| 31 | MCM7 |  |  | 4435 | -0.008 | -0.3831 | No |
| 32 | PLIN2 |  |  | 4478 | -0.008 | -0.3872 | No |
| 33 | EFNB1 |  |  | 4526 | -0.009 | -0.3918 | No |
| 34 | CDCA5 |  |  | 4577 | -0.010 | -0.3966 | No |
| 35 | CCNB2 |  |  | 4736 | -0.013 | -0.4136 | No |
| 36 | UHRF1 |  |  | 4792 | -0.014 | -0.4187 | No |
| 37 | ZFHX4 |  |  | 4941 | -0.017 | -0.4341 | No |
| 38 | CENPM |  |  | 5089 | -0.020 | -0.4492 | No |
| 39 | C21orf58 |  |  | 5096 | -0.020 | -0.4480 | No |
| 40 | C19orf48 |  |  | 5117 | -0.021 | -0.4484 | No |
| 41 | GTSE1 |  |  | 5195 | -0.022 | -0.4553 | No |
| 42 | BUB1 |  |  | 5377 | -0.026 | -0.4738 | No |
| 43 | H2AFX |  |  | 5451 | -0.027 | -0.4797 | No |
| 44 | BUB3 |  |  | 5807 | -0.034 | -0.5175 | No |
| 45 | KNSTRN |  |  | 5875 | -0.036 | -0.5219 | No |
| 46 | H2AFZ |  |  | 6079 | -0.040 | -0.5416 | No |
| 47 | SPAG5 |  |  | 6146 | -0.041 | -0.5455 | No |
| 48 | SFRP2 |  |  | 6191 | -0.042 | -0.5466 | No |
| 49 | CENPN |  |  | 6194 | -0.042 | -0.5430 | No |
| 50 | AURKB |  |  | 6215 | -0.043 | -0.5413 | No |
| 51 | CDKN3 |  |  | 6291 | -0.044 | -0.5459 | No |
| 52 | CDCA4 |  |  | 6310 | -0.045 | -0.5439 | No |
| 53 | GINS2 |  |  | 6461 | -0.049 | -0.5567 | No |
| 54 | PHGDH |  |  | 6659 | -0.054 | -0.5744 | No |
| 55 | MKI67 |  |  | 6750 | -0.057 | -0.5795 | No |
| 56 | MCM5 |  |  | 6765 | -0.058 | -0.5758 | No |
| 57 | PRC1 |  |  | 6918 | -0.062 | -0.5876 | No |
| 58 | GLI3 |  |  | 7020 | -0.066 | -0.5932 | Yes |
| 59 | TACC3 |  |  | 7022 | -0.066 | -0.5873 | Yes |
| 60 | BIRC5 |  |  | 7024 | -0.066 | -0.5814 | Yes |
| 61 | TMEM98 |  |  | 7034 | -0.066 | -0.5764 | Yes |
| 62 | CKAP2L |  |  | 7065 | -0.067 | -0.5737 | Yes |
| 63 | KIF22 |  |  | 7098 | -0.068 | -0.5712 | Yes |
| 64 | FEN1 |  |  | 7218 | -0.071 | -0.5783 | Yes |
| 65 | MIS18BP1 |  |  | 7253 | -0.073 | -0.5756 | Yes |
| 66 | HES1 |  |  | 7301 | -0.074 | -0.5741 | Yes |
| 67 | ZWINT |  |  | 7370 | -0.077 | -0.5749 | Yes |
| 68 | TOP2A |  |  | 7430 | -0.079 | -0.5745 | Yes |
| 69 | RRM1 |  |  | 7491 | -0.081 | -0.5740 | Yes |
| 70 | SPC25 |  |  | 7522 | -0.082 | -0.5699 | Yes |
| 71 | HAT1 |  |  | 7538 | -0.083 | -0.5640 | Yes |
| 72 | MCM3 |  |  | 7597 | -0.086 | -0.5628 | Yes |
| 73 | KIF11 |  |  | 7619 | -0.087 | -0.5572 | Yes |
| 74 | CDCA3 |  |  | 7631 | -0.087 | -0.5505 | Yes |
| 75 | HIST1H4C |  |  | 7641 | -0.088 | -0.5435 | Yes |
| 76 | GMNN |  |  | 7651 | -0.088 | -0.5364 | Yes |
| 77 | DEPDC1B |  |  | 7692 | -0.091 | -0.5327 | Yes |
| 78 | NDC80 |  |  | 7734 | -0.093 | -0.5289 | Yes |
| 79 | MCM4 |  |  | 7900 | -0.101 | -0.5386 | Yes |
| 80 | PBK |  |  | 7905 | -0.101 | -0.5298 | Yes |
| 81 | NUF2 |  |  | 7936 | -0.103 | -0.5238 | Yes |
| 82 | CCNB1 |  |  | 7958 | -0.104 | -0.5166 | Yes |
| 83 | ITGB3BP |  |  | 7959 | -0.104 | -0.5070 | Yes |
| 84 | RRM2 |  |  | 8001 | -0.107 | -0.5019 | Yes |
| 85 | KPNA2 |  |  | 8045 | -0.110 | -0.4968 | Yes |
| 86 | PCNA |  |  | 8061 | -0.111 | -0.4883 | Yes |
| 87 | CKS2 |  |  | 8077 | -0.112 | -0.4798 | Yes |
| 88 | MCM2 |  |  | 8099 | -0.114 | -0.4718 | Yes |
| 89 | CNTLN |  |  | 8150 | -0.117 | -0.4668 | Yes |
| 90 | CCNA2 |  |  | 8227 | -0.124 | -0.4642 | Yes |
| 91 | KIF15 |  |  | 8228 | -0.124 | -0.4528 | Yes |
| 92 | LDHA |  |  | 8261 | -0.127 | -0.4448 | Yes |
| 93 | FBXO5 |  |  | 8310 | -0.133 | -0.4381 | Yes |
| 94 | KIF2C |  |  | 8328 | -0.135 | -0.4276 | Yes |
| 95 | CKAP2 |  |  | 8330 | -0.136 | -0.4153 | Yes |
| 96 | CDCA7 |  |  | 8337 | -0.137 | -0.4034 | Yes |
| 97 | CLIC1 |  |  | 8343 | -0.137 | -0.3914 | Yes |
| 98 | CDK1 |  |  | 8351 | -0.139 | -0.3794 | Yes |
| 99 | ATAD2 |  |  | 8359 | -0.141 | -0.3673 | Yes |
| 100 | TPX2 |  |  | 8370 | -0.143 | -0.3554 | Yes |
| 101 | HMGB2 |  |  | 8388 | -0.145 | -0.3440 | Yes |
| 102 | CENPU |  |  | 8403 | -0.147 | -0.3321 | Yes |
| 103 | MAD2L1 |  |  | 8427 | -0.150 | -0.3211 | Yes |
| 104 | MELK |  |  | 8437 | -0.150 | -0.3083 | Yes |
| 105 | CLSPN |  |  | 8440 | -0.151 | -0.2947 | Yes |
| 106 | DLGAP5 |  |  | 8447 | -0.152 | -0.2814 | Yes |
| 107 | TAGLN2 |  |  | 8457 | -0.153 | -0.2684 | Yes |
| 108 | KIF23 |  |  | 8465 | -0.154 | -0.2550 | Yes |
| 109 | ORC6 |  |  | 8482 | -0.157 | -0.2424 | Yes |
| 110 | UBE2T |  |  | 8489 | -0.159 | -0.2285 | Yes |
| 111 | NUSAP1 |  |  | 8523 | -0.167 | -0.2170 | Yes |
| 112 | TUBB4B |  |  | 8554 | -0.173 | -0.2045 | Yes |
| 113 | PRR11 |  |  | 8580 | -0.182 | -0.1907 | Yes |
| 114 | DHFR |  |  | 8590 | -0.186 | -0.1747 | Yes |
| 115 | RCN1 |  |  | 8602 | -0.191 | -0.1584 | Yes |
| 116 | PAICS |  |  | 8653 | -0.214 | -0.1445 | Yes |
| 117 | ASPM |  |  | 8657 | -0.215 | -0.1250 | Yes |
| 118 | SMC2 |  |  | 8658 | -0.216 | -0.1052 | Yes |
| 119 | CENPK |  |  | 8666 | -0.220 | -0.0858 | Yes |
| 120 | CENPF |  |  | 8703 | -0.240 | -0.0679 | Yes |
| 121 | HELLS |  |  | 8720 | -0.260 | -0.0459 | Yes |
| 122 | UBE2C |  |  | 8748 | -0.286 | -0.0228 | Yes |
| 123 | SMC4 |  |  | 8764 | -0.307 | 0.0037 | Yes |
Table: GSEA details [plain text format]

  

Fig 2: RICHARDS\_ZHONG\_NATURE\_2018\_NPCS\_UPREG: Random ES distribution      
 Gene set null distribution of ES for **RICHARDS\_ZHONG\_NATURE\_2018\_NPCS\_UPREG**
